# Supplementary material for: Hybrid Models and Biological Model Reduction with PyDSTool
Source: PLoS Comput Biol. 2012 Aug 9;8(8):e1002628. doi: 10.1371/journal.pcbi.1002628 (PMC3415397; doi:10.1371/journal.pcbi.1002628)
Supplement: Text S4 — Complete source code for the PyDSTool package (version 0.88.120504). Includes API documentation and help files linking to web pages. This file is identical to the current public release on Sourceforge.net. (ZIP) [file pcbi.1002628.s004.zip › PyDSTool/html/PyDSTool.Toolbox.optimizers.optimizer-pysrc.html]

xml version="1.0" encoding="ascii"?


PyDSTool.Toolbox.optimizers.optimizer


| Home | Trees | Indices | Help | PyDSTool |
| --- | --- | --- | --- | --- |

Package PyDSTool ::
Package Toolbox ::
Package optimizers ::
Package optimizer

[hide private]

[frames] | no frames]

# Source Code for Package PyDSTool.Toolbox.optimizers.optimizer

```
 1   
 2  # Matthieu Brucher 
 3  # Last Change : 2007-07-23 10:50 
 4   
 5  """ 
 6  Module containing the core optimizers 
 7   
 8  Optimizers : 
 9    - Optimizer 
10      - a skeletton for defining a custom optimizer 
11      - calls iterate until the criterion indicates that the optimization has converged 
12    - StandardOptimizer 
13      - takes a full step after a line search 
14    - StandardOptimizerModifying 
15      - takes a full step after a line search 
16      - modifies the resulting parameters 
17  """ 
18   
19  from standard_optimizer import * 
20  from standard_optimizer_modifying import * 
21   
22  optimizer__all__ = ['StandardOptimizer', 'StandardOptimizerModifying'] 
23   
24  __all__ = optimizer__all__ 
25
```

  


| Home | Trees | Indices | Help | PyDSTool |
| --- | --- | --- | --- | --- |

|  |  |
| --- | --- |
| Generated by Epydoc 3.0.1 on Fri May 4 15:24:15 2012 | http://epydoc.sourceforge.net |
